# Tunable Multi-switching in Plasmonic Waveguide with Kerr Nonlinear Resonator

**DOI:** 10.1038/srep15837

**Published:** 2015-10-29

**Authors:** Zhihui He, Hongjian Li, Shiping Zhan, Boxun Li, Zhiquan Chen, Hui Xu

**Affiliations:** 1 College of Physics and Electronics, Central South University, Changsha 410083, China; 2 College of Materials Science and Engineering, Central South University, Changsha 410083, PR China

## Abstract

We propose a nanoplasmonic waveguide side-coupled with bright-dark-dark resonators in our paper. A multi-oscillator theory derived from the typical two-oscillator model, is established to describe spectral features as well as slow-light effects in bright-dark-dark structures, and confirmed by the finite-difference time domain (FDTD). That a typical plasmon induced transparency (PIT) turns to double PIT spectra is observed in this waveguide structure. At the same time, multi-switching effects with obvious double slow-light bands based on double PIT are also discovered in our proposed structure. What’s more, dynamically tuning the multi-switching is achieved by means of filling Fabry-Perot resonators with the Kerr nonlinear material Ag-BaO. These results may have applications in all-optical devices, moreover, the multi-oscillator theory may play a guiding role in designing plasmonic devices.

PIT is a classical analogue of atomic electromagnetically induced transparency (EIT)^1^,^2^, which has attracted enormous attention because of its important applications in the fields of slow-light effects^3^,^4^, and integrated photonic devices^5^,^6^. The classical analogue of EIT observed in nanoscale plasmonic resonator systems were theoretically and experimentally demonstrated^7^,^8^,^9^,^10^,^11^. The single dark resonator has been studied in a variety of bright-dark systems^12^,^13^. Recently, Philippe Tassin *et al.*^14^ reported a two-oscillator model to demonstrate analogue of EIT peaks in metamaterials. He *et al.*^15^ introduced the two-oscillator theory model to describe the PIT in waveguide systems. The oscillator theory model can effectively discuss both transmission spectra and scattering parameters in normal bright-dark structures. However, there are few articles, which aimed at developing the two-oscillator theory model so as to investigate multiple dark resonators. Then, based on PIT, Lu *et al.*^16^ reported ultrafast all-optical switching in nanoplasmonic waveguides. Han *et al.*^17^ aimed at researching low-power and ultrafast all-optical tunable PIT in metal-dielectric-metal (MDM) waveguide side-coupled Fabry-Perot resonators systems. However, multi-switching effects based on double PIT are rarely studied in plasmonic waveguide structures. Furthermore, few comprehensive studies have been performed on dynamically tuning the multi-switching in nanoplasmonic waveguides.

In our paper, we provide a multi-oscillator theoretical description of PIT in single bright with multiple dark waveguide structures. And a bright-dark-dark waveguide structure is proposed to support this theory model. In our research, double PIT spectra are found in our proposed structure, followed by observation of the plasmonic multi-switching effects and double slow-light bands. Moreover, we can dynamically regulate the multi-switching which may has an application in digital optics through filling Fabry-Perot resonators with Kerr nonlinear materials.

## Analytic theory

We assume a nanoplasmonic waveguide side-coupled with single bright mode and multiple dark modes resonators. The first cavity is excited by the bus waveguide, so it is regarded as a bright mode. And the second cavity, which is excited by the first cavity, can be considered as a dark mode. The third cavity is excited by the second cavity and so on. In other words, the case where the *j*th cavity only has interaction with the (*j* − 1)th cavity and the (*j* + 1)th cavity. Here, we introduce an extended multi-oscillator theory derived from the typical two-oscillator model where *D*_*j*_ = 1 − *(ω*_*/*_*ω*_*j*_)^2^ − *iγ*_*j*_*(ω*_*/*_*ω*_*j*_)^14^. (*j* = 1,2…N). The *j*th resonator with the resonance frequency *ω*_*j*_ and the damping factor *γ*_*j*_ is described by the excitation *p*_*j*_*(ω)* (*j* = 1,2…N). The first cavity is also driven by the external force *f(ω)*. *κ*_*j*_ is the coupling strength between the *j*th cavity and the *(j* + 1)th cavity (*j* = 1,2…N-1). A coupled harmonic matrix equation can describe these systems


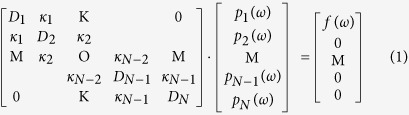


The matrix Eq. (1) can be solved as follows


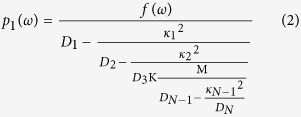


The electric current sheet with conductivity *σ*_*N*_ = *−iωp(ω)/f(ω)* is introduced to describe this effective response^15^. The conductivity *σ*_*N*_ in single bright mode with N-1 dark modes waveguide structures can be written as


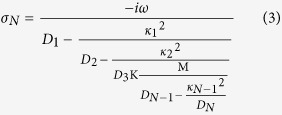


The transmission coefficient and the group index in the MDM waveguide system can be calculated in the following form^14^,^15^.


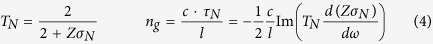


where *τ*_*N*_ is the group delay of single bright mode with N-1 dark modes waveguide structures^14^. *c* is the velocity of the light in vacuum, *l* = 700 nm is the length of the bus waveguide. *Z* = *β(w)w/ωε*_*0*_*ε*_*1*_^18^ is the wave impedance, where *ε*_*0*_ is the permittivity of vacuum, *ε*_*1*_ is the relative permittivity of the filled medium in resonators. *β(w)* is a propagation constant in MDM resonators.

## Simulation results and discussions

**Figure 1:**
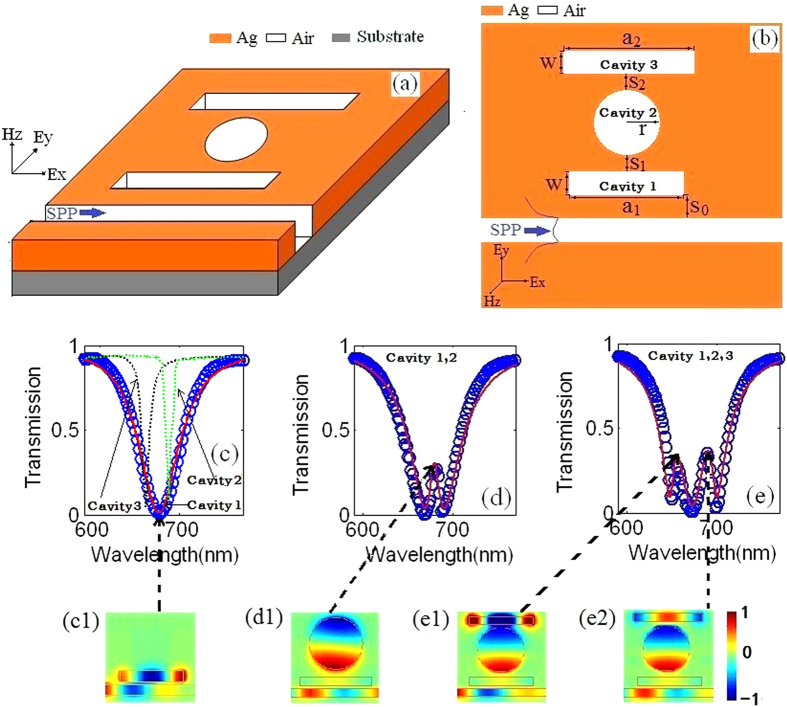
(**a**) Schematic of the bright-dark-dark nanoplasmonic waveguide. (**b**) Top view of this waveguide structure. (**c**–**e**) Transmission spectra of the plasmonic waveguide side coupled with cavity 1 (red solid line and blue circle line in (**c**)), cavity 2 (green dash line), cavity 3 (black dash line), cavity 1 and 2, cavity 1, 2 and 3 with S_0_ = 20 nm, S_1_ = 30 nm, S_2_ = 30 nm a_1_ = 400 nm, a_2_ = 430 nm and r = 165 nm. The blue circle is the simulation result, and the red line is the theory result. (**c1**) The magnetic field Hz at the dip when the bus waveguide side coupled with cavity 1. (**d1**) The magnetic field Hz at the peak when the bus waveguide side coupled with cavity 1 and 2. (**e1**,**e2**) The magnetic field Hz at the two peaks when the bus waveguide side coupled with cavity 1, 2 and 3.

Here, we provide a bright-dark-dark MDM waveguide as shown in Fig. 1(a,b). The frequency dependent optical property of the silver nanostructure is approximated by the Drude model^19^ *ε(ω)* = *ε*_*∞*_ − *ω*_*p*_^2^*/(ω*^2^ *+* *iωγ*_*p*_), with *ω*_*p*_ = 1.38 × 10^16^ s^−1^ is the bulk plasmon frequency, *ε*_*∞*_ = 3.7 and *γ*_*p*_ = 2.73 × 10^13^ s^−1^ represents the damping rate. The characteristic spectral responses of the structures are found by using the two-dimensional FDTD^20^ method with Δx = Δy = 5 nm. We set the light source at the entrance of the bus waveguide. A normalized screen is placed at the exit of the bus waveguide. The calculated domain is surrounded by perfectly matched layer absorbing boundary. The geometric parameters are set as follows: a_1_ = 400 nm, and the width of resonators and buswaveguide W = 50 nm. In this bright-dark-dark waveguide, the conductivity *σ*_*N*_ can be reduced as the conductivity *σ*_*3*_ in the following form


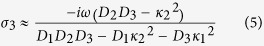


where *κ*_*1*_ is the coupling strength between the cavity 1 and cavity 2. *κ*_*2*_ is the coupling strength between the cavity 2 and cavity 3.

In order to verify the theoretical analysis above, we study transmission spectra of the nanoplasmonic waveguide side coupled with cavity 1, cavity 2, cavity 3, cavity 1 and 2, cavity 1, 2 and 3 as shown in Fig. 1(c–e), respectively. Figure 1(c) shows the transmission spectra when the bus waveguide side-coupled with cavity 1 (red solid line and blue circle line), cavity 2 (green dash line) and cavity 3 (black dash line), respectively. We can find that the thansmission spectra of cavity 1 is a wide-band superradiant state, so cavity 1 can be regarded as a bright mode^21^,^22^. Conversely, trasmission spectra of cavity 2 and cavity 3 are narrow-band subradiant states, thus, cavity 2 and cavity 3 are dark modes^21^,^22^. Then we can see a typical PIT in Fig. 1(d), however, what is interesting is that double PIT spectra appear in Fig. 1(e). To further illustrate the phenomenon mentioned above, we depict the magnetic field Hz. The magnetic field distribution Hz, which corresponds to the dip in Fig. 1(c), is plotted in Fig. 1(c1). We can find that the Hz is strongly limited in cavity 1. However, at the transmission peak in Fig. 1(d), cavity 2 which serves as a dark mode is strongly excited. Conversely, the strong excitation of the dark mode may suppress the oscillation of the bright mode in a destructive way. Therefore, a PIT peak occurs in Fig. 1(d). These discriptions about PIT in brigh-dark mode structures have been reported in recent researches^23^,^24^. Finally, we describe the magnetic field distribution Hz at peaks when the bus waveguide side-coupled with cavity 1, 2 and 3. We can detect that cavity 2 and cavity 3 are excited in Fig. 1(e1. Just like the magnetic filed distribution Hz in Fig. 1(d1), there is almost no energy localizes in cavity 1. And the interaction between the bright mode and two dark modes results in the double PIT phenomenon. This research verifies the correctness of the extended multi-oscillator model.

**Figure 2:**
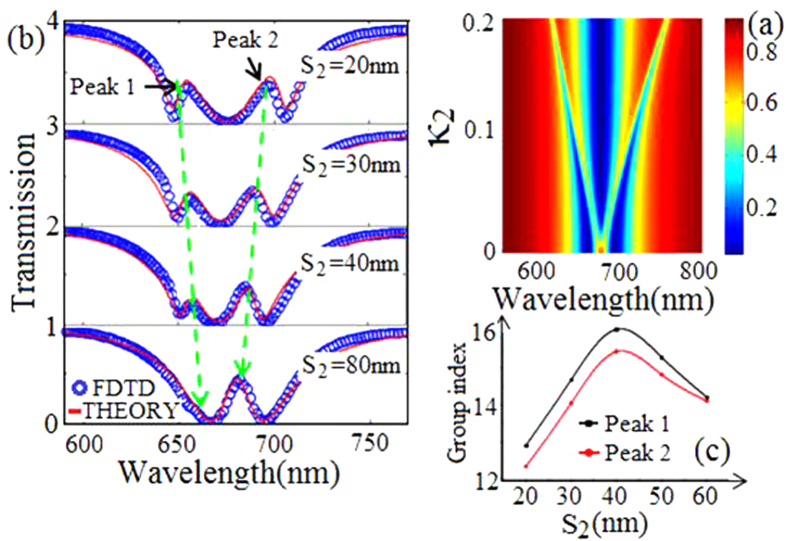
(**a**) Evolution of the transmission spectra versus κ_2_ and λ. (**b**) Transmission spectra with different coupling distance S_2_, the other parameters are S_0_ = 20 nm, S_1_ = 30 nm, a_2_ = 430 nm and r = 165 nm. (**c**) The group index at the transmission peak 1 (black marked line) and peak 2 (red marked line) with different S_2_.

We investigate transmission characteristics and slow-light effects as a function of the coupling strength κ_2_ in our proposed structure for further research. We plot the transmission spectra with parameters damping factors γ_1_ = 0.01, γ_2_ = 0.005, γ_3_ = 0.008, coupling strength κ_1_ = 0.05, and the κ_2_ range from 0 to 0.2 in Fig. 2(a). We recognize the typical feature of PIT when coupling strength κ_2_ is very weak. Interestingly, Fig. 2(a) shows double PIT spectra as coupling strength κ_2_ increases. The transmission spectra are shown in Fig. 2(b) as a function of S_2_. The blue circles are simulation data, while the red lines are theory data. The calculated results are in well agreement with FDTD simulations. Equation (5) can be reduced in well-established form for two-oscillator model^15^ by assuming that cavity 3 is far from cavity 2. Thus a typical feature of PIT is observed in Fig. 2(b) when S_2_ = 80 nm. In addition, transmission peak 1 and peak 2 are close to the center in Fig. 2(a,b) as the coupling strength κ_2_ weakens. Then slow-light effects are investigated in this waveguide structure as shown in Fig. 2(c). At the peak 1 (black marked line) and the peak 2 (red marked line), the group index first increases and then decreases with the increasing of S_2_. To analyze the phenomenon above, cavity 2 and cavity 3 can be considered as a whole to play a role of a dark mode. When we increase S_2_, the centre of the whole dark mode will get far away from the bright mode. As a result, the energy coupling to the whole dark mode will be weakened. Thus, the group index increases with the increasing of S_2_. This conclusion can be found in the reported article^14^. However, as S_2_ increases, the impedance in the whole dark mode also increases, so the damping factor in the whole dark mode increases. The group index decreases with the increasing of the damping factors in the dark mode^14^. Therefore, the group index decreases with the increasing of S_2_. To conclusion, when S_2_ ranges from 0 to 40 nm, the coupling strength between the bright mode and the whole dark mode is the most primary factor for group index. However, when S_2_ > 40 nm, the most primary factor for group index is not the coupling strength but damping factor in the whole dark mode. As a consequence of this, the group index first increases and then decreases in Fig. 2(c). This research provides a convenient tuning of double PIT, and may guarantee a wider application in integrated plasmonic devices.

**Figure 3:**
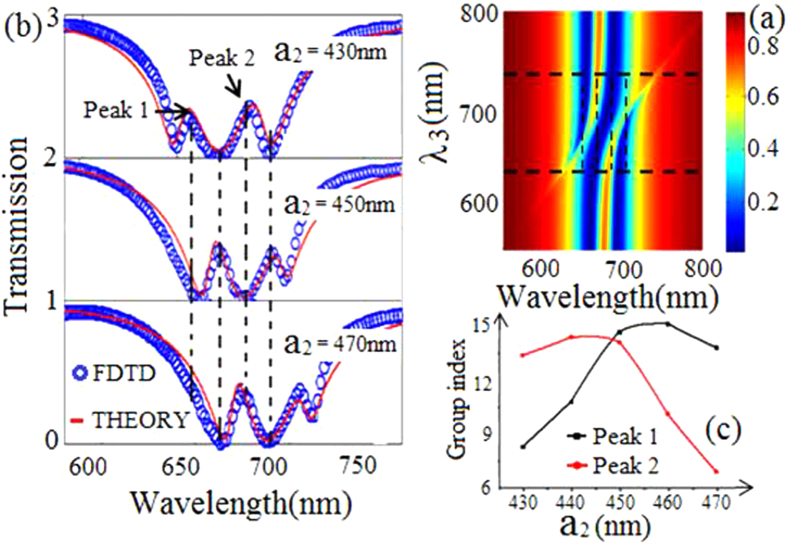
(**a**) Evolution of transmission spectra versus resonance wavelength λ_3_ and incident wavelength λ. (**b**) Transmission spectra with different length a_2_, the other parameters are S_0_ = 20 nm, S_1_ = S_2_ = 30 nm and r = 165 nm. (**c**) The group index at the transmission peak 1 (black marked line) and peak 2 (red marked line) for different a_2_.

Next, we study transmission amplitudes when the resonance wavelength λ_3_ of cavity 3 increases in Fig. 3(a). The parameters λ_1_ = λ_2_ = 670 nm, and λ_3_ ranges from 550 nm to 800 nm. We can see the typical PIT when λ_3_ > 740 nm or λ_3_ < 630 nm. The interesting thing, however, is that Fig. 3(a) exhibits the double PIT when 630 nm < λ_3_ < 740 nm. Theoretical and simulative transmission spectra are plotted in Fig. 3(b) with different a_2_ in our proposed structure. The double PIT spectra are observed in the transmission spectra. As a_2_ increases from 430 nm to 470 nm, we can see the two transparency peaks show red shift. And this phenomenon corresponds with the theoretical results. That is because changing a_2_ not only tunes resonance frequency of cavity 3, but also slightly affects the coupling between cavities. Then, the slow-light effects in this waveguide structure are investigated as shown in Fig. 3(c). We can find that the group index at the peak 1 and peak 2 first increases and then decreases with the increasing of a_2_. The reason is that the largest group index often appears when resonance wavelength of a bright mode is equal to that of a dark mode^14^. So the group index increases when a_2_ ranges from 430 nm to 460 nm, while decreases when a_2_ > 460 nm. This result provides a convenient tuning of slow-light effects.

**Figure 4:**
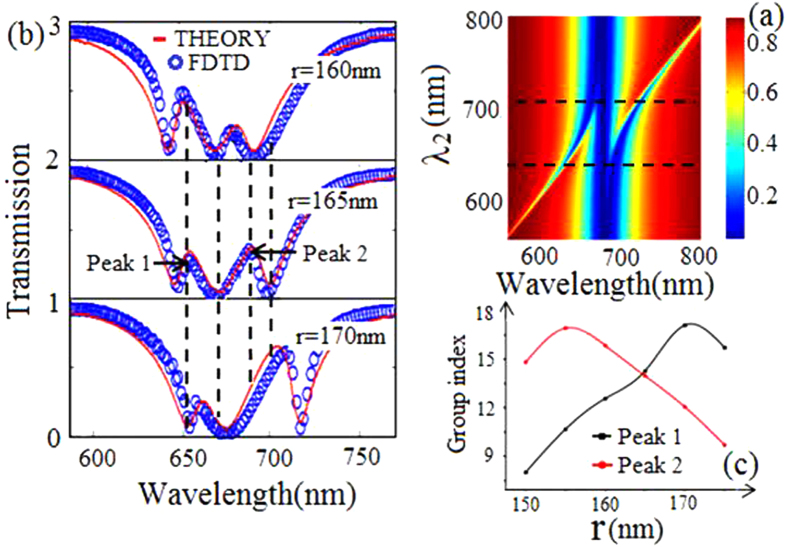
(**a**) Evolution of transmission spectra versus resonance wavelength λ_2_ and incident wavelength λ. (**b**) Transmission spectra with different radius r, the other parameters are S_0_ = 20 nm, S_1_ = S_2_ = 30 nm and a_2_ = 430 nm. (**c**) The group index at the transmission peak 1 (black marked line) and peak 2 (red marked line) for different r.

At last, we investigate transmission spectra with the increasing of the resonance wavelength λ_2_ of cavity 2. The parameters λ_1_ = λ_3_ = 670 nm, and λ_2_ increases from 550 nm to 800 nm. Figure 4(a) shows the double PIT when 655 nm < λ_2_ < 708 nm. The transmission spectra of FDTD and the theoretical results, which fit well with each other, are plotted in Fig. 4(b) with different r. The two transparency peaks show red shift with the increasing of r. Then, we work around slow-light effects and get the following results: the group index first increases and then decreases with the increasing of r at peak 1 and peak 2 as shown in Fig. 4(c).

In addition, we can see that the transmission spectra have switching effects at 660 nm, 676 nm, 692 nm and 701 nm with a_2_ = 430 nm and 450 nm as shown in Fig. 3(b). Similar phenomenon can be found in Fig. 4(b). According to this study, we can predict that our proposed structure may achieve function of the plasmonic multi-switching. However, the switching based on changing the geometric parameters is a static one. In order to make this multi-switching tunable, we introduce the following research.

## Dynamic tunable Multi-switching effects

As resonant cavities in our proposed structure can be regarded as Fabry-Perot optical resonant cavities^25^ with *m·λ* = 2*a*_*i*_*·Re(n*_*eff*_) (where λ is the resonance wavelength of resonator, *m* is the order of resonance mode, *a*_*i*_ is the length of cavities, and *n*_*eff*_ is the effective refractive index of cavity), not only can we change the dimension, but we can also change the effective refractive index neff to tune the resonance frequency. If we fill the Fabry-Perot resonators with Kerr nonlinear materials, the resonance wavelength in bright and dark modes can be actively tuned by changing the pump intensity. At this point, our proposed structure may realize the function of dynamic tunable multi-switch effects^16^. Furthermore, our plasmonic waveguide with Kerr nonlinear resonators can be used for all-optical switches. Since slow-light effects can enhance energy in local area, it can reduce switching energy in all-optical switches^26^,^27^.

**Figure 5:**
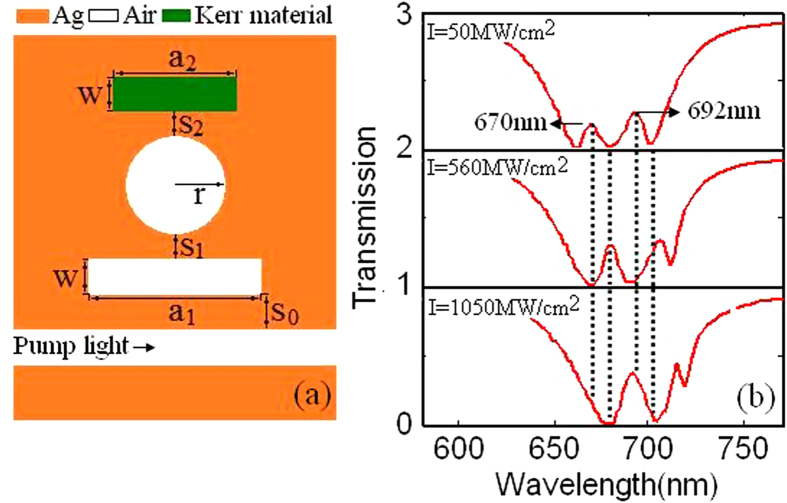
(**a**) Schematic of the bright-dark-dark plasmonic waveguide with cavity 3 filled with the Kerr nonlinear material. The parameters are S_0_ = 20 nm, S_1_ = 30 nm, S_2_ = 30 nm, W = 50 nm, a_1_ = 400 nm, a_2_ = 300 nm and r = 165 nm. (**b**) Transmission spectra of plasmonic waveguide side coupled with Kerr nonlinear resonator as a function of pump light intensity.

As you can see in Fig. 5(a), the plasmonic waveguide is side-coupled with cavity 3 filled with a kind of Kerr nonlinear material whose dielectric constant *ε*_c_ depends on the intensity of electric field |*E*|^2^: *ε*_c_ = *ε*′ *+* *χ*^(3)^|*E*|^2^. The value of linear dielectric constant *ε*′ is 2.0. The Kerr nonlinear material is assumed to be Ag-BaO, and its third-order nonlinear is *χ*^(3)^  = 4.8 × 10^−10^ esu. Transmission spectra as a function of the pump light intensity are shown in Fig. 5(b). Comparing Fig. 5(b) with Fig. 3(b), we can find that tuning pump light intensity and changing geometric parameters can achieve the same effect. We assume that the transmission larger than 0.15 is regarded as switch-on, and considered as 1 in digital circuits. On the contrary, switch-off can be considered as 0. We list the transmission ratios, the switch-on/off and the binaries at 670 nm, 680 nm, 692 nm and 700 nm with the pump light intensity I = 50 MW/cm^2^ and 560 MW/cm^2^ in Table 1, respectively. We can find that the two status of switches are the polar opposite when I = 50 MW/cm^2^ and 560 MW/cm^2^. These results may be applied to optical switch devices. Here, we can also achieve the binary array (1 0 1 0) at 670 nm, 680 nm, 692 nm and 700 nm when a_2_ = 430 nm, and the binary array (0 1 0 1) when a_2_ = 450 nm, as it is observed in Table 1.

**Figure 6:**
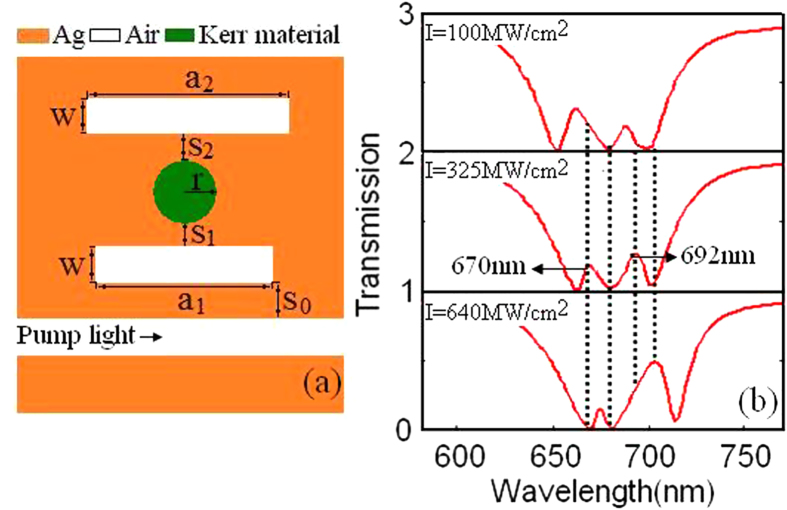
(**a**) Schematic of the bright-dark-dark plasmonic waveguide with cavity 2 filled with the Kerr nonlinear material. The parameters are S_0_ = 20 nm, S_1_ = 30 nm, S_2_ = 30 nm, W = 50 nm, a_2_ = 430 nm and r = 105 nm. (**b**) Transmission spectra of plasmonic waveguide side coupled with Kerr nonlinear resonator as a function of pump light intensity.

Finally, as Fig. 6 shows, we investigate transmission spectra with the increasing of pump light intensity in the plasmonic waveguide side-coupled with cavity 3 filled with the Kerr nonlinear material. Just as we expected, the transmission spectra in Fig. 6(b) are similar to those in Fig. 4(b). Then we list the transmission ratios, the switch-on/off and the binaries at 670 nm, 680 nm, 692 nm and 700 nm with the pump light intensity I = 100 MW/cm^2^ and 640 MW/cm^2^ in Table 2, respectively. Here, we can get the binary array (1 0 0 0) at 670 nm, 680 nm, 692 nm and 700 nm when I = 100 MW/cm^2^, and the binary array (0 0 1 1) when I = 640 MW/cm^2^. What is interesting is that we can obtain binary arrays (0 0), (0 1), (1 0) and (1 1) at 670 nm and 692 nm with different parameters summarized in Tables 1 and 2. These binary arrays may have an application in digital optical circuits.

## Conclusions

To summarize, we propose a multi-oscillator theory to describe PIT in a nanoplasmonic waveguide side-coupled with bright-dark-dark resonators in our paper. On the base of PIT, through the method of changing geometric parameters, multi-switching effects with obvious double slow-light bands are realized. However, it is far more convenient to dynamically tune the multi-switching by means of filling Fabry-Perot resonators with Kerr nonlinear material. Our research may pave the way for designing plasmonic switches.

## Methods

The frequency dependent optical property of the silver nanostructure is approximated by the Drude model *ε(ω)* = *ε*_*∞*_ − *ω*_*p*_^2^*/(ω*^2^ *+* *iωγ*_*p*_), with *ω*_*p*_ = 1.38 × 10^16^ s^−1^ is the bulk plasmon frequency, *ε*_*∞*_ = 3.7 and *γ*_*p*_ = 2.73 × 10^13^ s^−1^ represents the damping rate. The characteristic spectral responses of the structures are found by using the two-dimensional FDTD method with Δx = Δy = 5 nm. We set the light source at the entrance of the bus waveguide. A normalized screen is placed at the exit of the bus waveguide.The calculated domain is surrounded by perfectly matched layer absorbing boundary. We choose Meep as our FDTD simulation software developed at MIT. And the simulation parameters have been given in our paper.

## Additional Information

**How to cite this article**: He, Z. *et al.* Tunable Multi-switching in Plasmonic Waveguide with Kerr Nonlinear Resonator. *Sci. Rep.* **5**, 15837; doi: 10.1038/srep15837 (2015).

## Figures and Tables

**Table 1:** The plasmonic multi-switch at transmission peaks and dips in plasmonic waveguide with cavity 3 filled with Ag-BaO.

λ(nm)	I = 50 MW/cm^2^			I = 560 MW/cm^2^		
	Transmission	on/off	Binary	Transmission	on/off	Binary
670	0.180	on	1	0.006	off	0
680	0.017	off	0	0.355	on	1
692	0.270	on	1	0.028	off	0
700	0.035	off	0	0.324	on	1

**Table 2:** The plasmonic multi-switch at transmission peaks and dips in plasmonic waveguide with cavity 2 filled with Ag-BaO.

λ(nm)	I = 100 MW/cm^2^			I = 640 MW/cm^2^		
	Transmission	on/off	Binary	Transmission	on/off	Binary
670	0.210	on	1	0.009	off	0
680	0.020	off	0	0.017	off	0
692	0.045	off	0	0.248	on	1
700	0.050	off	0	0.493	on	1
